# Aerobic exercise and Alzheimer’s disease blood‐based biomarkers

**DOI:** 10.1002/alz.088040

**Published:** 2025-01-09

**Authors:** Kirk I. Erickson

**Affiliations:** ^1^ AdventHealth, Orlando, FL, USA; University of Pittsburgh, Pittsburgh, PA, USA; AdventHealth Research Institute, Neuroscience, Orlando, FL USA

## Abstract

A greater amount of physical activity is associated with a reduced risk of developing dementia, including Alzheimer’s disease. Engaging in physical activity affects several pathways that are associated with memory impairments including hippocampal integrity, resting state networks, and metabolic function. Further, exercise interventions are effective at improving various measures of brain health in late adulthood that overlap with the cognitive functions that these brain regions support. Yet, there remains a poor understanding of the mechanisms that link physical activity and exercise behaviors to improvements in brain health outcomes and reduced risk of developing dementia. There is also significant heterogeneity in this literature that could likely be explained by factors that moderate the impact of exercise on brain health. In this presentation we will discuss recent research on exercise and brain health in late adulthood and will discuss the evidence for several levels of mechanisms and moderators of these effects including examining the impact of exercise interventions on Alzheimer’s disease blood‐based biomarkers. Preliminary results will be described from a multi‐site, 12‐month, 3x per week supervised, moderate‐intensity, Phase III randomized clinical trial of aerobic exercise in 648 cognitively normal adults between 65‐80 years of age. Blood samples were taken before and after the completion of the intervention as well as a comprehensive cognitive battery and PET amyloid. The results from this aerobic exercise intervention will be discussed including the impact of the intervention on cognitive performance and the role that changes in Alzheimer’s disease blood‐based biomarkers play in relation to functional outcomes. Overall, physical activity and exercise behaviors are important modifiable lifestyles that carry significant consequences for learning, memory, and Alzheimer’s disease risk in late adulthood.

